# "The Hospital" Nursing Mirror

**Published:** 1897-06-12

**Authors:** 


### "The Hospital" Nursing Mirror.

Being the Nursing Section of "The Hospital."

[Contributions for this Section of "The Hospital" should be addressed to the Editor, The Hospital, 28 & 29, Southampton Street, Strand, London, W.C., and should have the word "Nursing" plainly written in left-hand top corner of the envelope.]

### News from the Nursing World.

### THE QUEEN'S COMMEMORATION.

A meeting of the Executive Committee of the Queen's Commemoration Fund in aid of the Jubilee Institute for Nurses was held last week at Grosvenor House by permission of the Duke of Westminster, its chairman. The Marquis of Lothian, the Countess of Selborne, Lord Reay, Sir Arthur Arnold, Mr. Bancroft, and the hon. secretaries, Mr. Harold Boulton and Mr. Ernest Flower, M.P., were amongst those who attended the meeting. The fund has now reached something over £52,000, and a promising report of the organisation in Scotland was given by Lord Reay. Statements as to the progress of Princess Henry of Pless's collection and the Children's Section were made. A large amount of money is expected from the latter source.

### COLONIAL NURSING ASSOCIATION.

Mr. Bancroft gave his reading of Dickens' "Christmas Carol" at the Imperial Institute in aid of the Colonial Nursing Association on Thursday, June 3rd. The East Conference Room, where the reading took place, was crowded, amongst those present being Lord and Lady Loch, Lord Stanmore, the Dowager Lady Westbury, Miss Chamberlain, and Sir H. H. and Lady Johnston. Lord Loch, who is hon. secretary of the association, took the opportunity of opening the proceedings to explain something of its object and work, and of the need for skilled nursing in the Crown Colonies which it was successfully attempting to meet. The reading was, as always, much appreciated, and at its conclusion the chairman announced that the association had benefited to the amount of over £250 by Mr. Bancroft's kindness.

### GODALMING NURSING ASSOCIATION.

A very successful American sale was held on the recreation ground at Godalming the other day, with the object of providing appliances for the nursing institution. Everyone worked with a will, and the result was altogether satisfactory, the end of the day seeing empty stalls and a profit of some £54. Everything was given for the tea, and the ground lent by the recreation club free of charge, so that expenses amounted to very little.

### UNWISE ADVICE.

A sanguine writer in a west country paper lately took upon himself to advise "nurses of experience" to emigrate to other lands, assuring them that a "great field for enterprise" lay before them in Africa, "especially, perhaps, in Johannesburg." We have addressed many a word of warning to nurses on this subject, and in case any may feel themselves inspired by the brilliant imaginings of the writer quoted above to "take their courage in both hands and go out alone, armed only with their skill, and, if possible, a letter of introduction to local doctors," it may be well to repeat once more our conviction that no nurse should dream of leaving England on any such doubtful quest. The strictest inquiries should be made from reliable quarters before starting off to foreign countries, and very little credence given to statements that fortunes are awaiting private nurses in Africa or anywhere else. Experience tells quite another tale.

### THE NURSING INSTITUTE, READING.

Messrs. Sutton and Sons, the well-known seed merchants, have conferred a most generous and timely favour on the town of Reading. It was decided some time since that the establishment of an efficient district nursing system should commemorate the Jubilee in Reading, and Messrs. Sutton have now offered a house to the Jubilee Nursing Committee for the proposed institution for a term of five years rent free. A meeting was held to discuss this kind offer, which was ultimately accepted with very hearty thanks. The house is in the Forbury, and will conveniently accommodate five nurses and a superintendent, the staff with which the institute is to be started. Any necessary alterations will be carried out at the owners' expense.

### STAFFORDSHIRE INSTITUTION FOR NURSES.

The 25th annual report of the Staffordshire Institution for Nurses is a particularly interesting one, giving a history of growth and progress from 1872, when five nurses, under the superintendence of Miss Sarah Harding, were established in a building erected in the grounds of the North Staffordshire Infirmary by the late Sir Smith Child in memory of his son. In 1877 a new home was completed, the number of nurses having risen to 18, with two probationers. In 1889 the home was enlarged, and in 1893 a cottage built for the special use of nurses laid aside by illness. Now the staff numbers 120—83 private nurses, with 22 district nurses and 15 probationers, the earnings of the former amounting to £4,800 3s. 8d. last year. A bonus of £332 from last year's balance was divided amongst the nurses this spring. During the year, in response to a special request from the nurses, a distinctive badge has been adopted, consisting of a Staffordshire knot, made in silver, with the initials S.N.I. A gold one was presented by the nurses to Miss Mary Shirley, the lady superintendent, "as a token of their affectionate regard."

### THE GRIEVANCES OF ASYLUM ATTENDANTS.

The *Daily Graphic* has been rendering good service to asylum workers by calling attention to the many very real "grievances" which beset them in their hard and trying lives. The account given by a correspondent of the circumstances under which the attendants are expected to take their meals in the wards shows that in many asylums there is an utter lack of even elementary comfort for the nurses and attendants. The matron of "a lunatic asylum in the metropolitan area," who was interviewed on the matter, spoke no less than the hard truth when she said that it was "almost impossible" for the agitation for reform to come from the workers themselves. The fate of reformers is generally a hard one, and those who venture to expose abuses know that they do so at the expense of future promotion—a serious consideration. Undoubtedly "the question is a public one," as the matron in question pointed out; and if the outside public would visit the county asylums and inquire into the question for themselves, surely some influence might be brought to bear in proper quarters which would hasten on improvement.

### QUEEN'S COMMEMORATION IN LIVERPOOL.

The committee of the Diamond Jubilee Commemoration Scheme in Liverpool are making a further appeal for the District Nursing Fund, and holding weekly meetings. Contributions at present amount to £15,000, but it is desired to increase this sum to at least £30,000 before the Fund closes.

### GENERAL HOSPITAL, BIRMINGHAM.

An interesting little ceremony took place in the board-room of the General Hospital, Birmingham, on Friday afternoon last, when, at an informal gathering of the resident and nursing staffs, the Rev. Stanley G. Collier, M.A., was presented with a handsome travelling clock, on his retirement from the chaplaincy of the hospital, which he had held for the past four years. Dr. J. Steed (one of the house physicians) made the presentation, referring to the cordial relationship which had existed between Mr. Collier and the members of the resident and nursing staffs, and expressing the regret of all that the state of Mr. Collier's health necessitated his resignation, and their heartiest good wishes for his early restoration to health.

### PREMATURE PROMOTION.

It is to be hoped that the Birkenhead Guardians will pause and consider well before they deliberately set aside their own rules for the workhouse infirmary and appoint a probationer of 22, with one year and eight months' experience, as charge nurse over an infirmary of 240 beds. By the rules probationers are received at the infirmary for three years' training, no certificates being given under that period, and one specially provides that no nurse shall be regarded either as assistant or as charge nurse unless she has served for the full three years; yet when the Infirmary Committee recommend that a charge nurse be advertised for, a number of the guardians actually urge the appointment to this important post of a very young probationer, who has not completed two years' training. Dr. Martin pointed out that it was a duty to the patients and to the public that an experienced nurse should be selected to fill such a post, and that it was a serious matter to place a "probationer in nursing" over an infirmary of 240 beds, with charge of training probationers. The committee consented to take back the resolution for further consideration, but they will be indeed ill-advised if they give way to the guardians on the point and make this most improper appointment.

### A DAY OF REST FOR LONDON NURSES.

On Thursday, the 3rd of this month, the nurses of the metropolitan hospitals and nursing institutions were invited by Canon Burnside to spend a day in the country at Hertingfordbury. The object in view was not only to afford some relaxation from work, but to exalt the Christian aspect of the ministry of nursing. There was a large attendance. The day commenced with a celebration of the Holy Communion in the parish church at half-past ten, at which the Bishop of Wakefield was celebrant, assisted by the chaplains of St. Bartholomew's, St. George's, and Westminster Hospitals, and the rector. Services were also held at twelve o'clock and three, the addresses throughout the day being given by the Bishop of Wakefield. At half-past one the nurses were entertained at luncheon, and after evensong, by the kindness of the Countess Cowper, they were invited to Pansanger, where tea was provided for them, and they afterwards had the opportunity of seeing the beautiful collection of pictures, and wandering through the gardens and park before going home. The Rev. A. G. Locke, of St. George's Hospital, acted as secretary, making all arrangements for the journey of the nurses from London, and to him many thanks are due.

### UNFIT FOR HUMAN HABITATION.

At last the old Camberwell Workhouse in Havil Street is to be pulled down, as should have been done long ago. It has taken a most serious report from Dr. Partridge, chairman of the Infirmary Visiting Committee, to bring about this result. Dr. Partridge stated that three of the medical officers were ill with quinsy and other ailments, while the nurses were suffering from headaches and sore throats, and the sanitary conditions of the building rendered it unfit for patients and officers alike. Rats were running about wholesale, nurses and patients being kept awake by them half the night. "The place was simply a fever den, and if gentlemen sent poor people to such a place they might be guardians of the poor, but they were also licensed murderers of the poor." Strong words, but surely justified by facts. It appears that the Local Government Board condemned the building long ago, but when the new infirmary became overcrowded, the Board actually yielded to the guardians' request, and certificated this wretched place for the accommodation of 100 patients, though "from the very first the occupants had to live in a horrible atmosphere, and illness was prevalent." It is at least satisfactory to learn that now a temporary iron building is to be put up for 120 patients, and a house to be rented for the accommodation of 16 nurses, but it is simply iniquitous that such a condition of things could be allowed to go on at all. Even if guardians have no consciences, something might be expected from Local Government Board inspectors. In the present case, by the testimony of Dr. Partridge, the lives of the medical officers, the nurses, and the patients have been ruthlessly endangered.

### SHORT ITEMS.

Miss Annie Lloyd, of the Women's Hospital, Birmingham, has successfully passed the Assistants' Examination of the Society of Apothecaries.—A satisfactory report was presented at the annual meeting of the Ruthin and District Nursing Association, the employment of another nurse and the extension of the district worked by the society being advocated by Colonel Cornwallis West. — The North Riding Nursing Association has passed a resolution calling upon the clergy of all denominations throughout the Riding to have a collection in their churches and chapels for the benefit of the association.—Mr. Bancroft has offered to give his reading of the "Christmas Carol," on behalf of the Cardiff and Swansea hospitals, in the autumn, an offer which has been gratefully accepted.—Her Royal Highness the Princess Christian of Schleswig-Holstein has graciously consented to become the President of the Society of Chartered Nurses. The Society, which was founded to supply to the public thoroughly competent private nurses, is under the management of a professional committee, and being conducted on co-operative principles, secures to each nurse the full remuneration of her work.

### Pharmacy and Dispensing for Nurses.

By C. J. S. Thompson.

XIII.—POWDERS—GARGLES—CACHETS—CAPSULES —TABLETS — ENEMAS — HYPODERMIC INJECTIONS.

In powders we have another method of administering medicine in a dry form. They are usually directed to be taken dry, by being placed on the tongue, or dissolved or stirred in a little water. They may consist of a single drug or a mixture of several, each dose being divided and wrapped separately in paper. In the latter case, the ingredients, after being weighed, must be placed in a mortar, reduced to fine powder, and well mixed together. The requisite number of pieces of paper having been spread out on the counter, the total amount for each powder must be weighed separately, and then neatly folded in the usual manner. The quantities should never be guessed. Glazed white paper of good quality should always be used for wrapping powders, and the utmost neatness observed in folding, so each shall be exactly similar in shape and size. Drugs of a volatile nature should be folded in tinfoil or waxed paper, and enclosed in white paper afterwards.

Gargles.—The gargle is the term applied to a liquid medicine used as an application for affections of the mouth, palate, and fauces. Gargles are usually composed of salts in solution, combined with certain astringent preparations, or glycerine. They rarely present any difficulty to the dispenser, and are prepared in a similar manner to mixtures. Chlorate of potassium is very frequently met with as an ingredient in gargles. In dissolving it, the use of the mortar is necessary. Any portion undissolved must also be placed in the bottle, which should be labelled with directions to shake. The following gargle is frequently prescribed with the object of administering chlorine in solution, the gas being liberated by the action of the hydrochloric acid on chlorate of potassium. ℞ Potass chlorat., ʒ i.; acid hydrochloric, ♏ xvi.; glycerine pur., ℥ i.; Aquæ ad., ℥ viii. Misce; fiat gargar. In dispensing this prescription, the chlorate of potassium should be placed in the bottle first, and the hydrochloric acid added to it. Cork the bottle loosely, and allow the chlorine to evolve. When the bottle is apparently full of the yellowish gas, add about four ounces of water and shake well for some time, then add the glycerine, and after further shaking for several minutes, make up to the required quantity with water.

Cachets.—The method of administering insoluble and unpalatable drugs in an envelope composed of thin wafer paper called a cachet, has been largely employed on the Continent, and France in particular, for a considerable time. Within the last few years this mode of administration has become very popular in our own country. The cachets in general use are those of Morstadt or Finot, and consist of two circular bowl-shaped discs with flat edges. The drug to be enclosed should be reduced to fine powder, and then packed into the bowl part of one of the discs. The edges of another disc having been slightly moistened, it should be placed over that containing the powder, the edges or rims being pressed together. Several handy machines are made, by means of which a dozen or more cachets can be filled and sealed with very little trouble. In this form the most nauseous drugs may be easily swallowed by the most fastidious patient.

Capsules.—The capsule is another method of administering both solid and liquid medicines which has become very popular with prescribers. For liquid medicines, flexible capsules of gelatine are made to hold from five to thirty minims. They are usually egg-shaped in form, with an aperture at one end, by means of which they are filled with the aid of a pipette, or small syringe. They are sealed by passing a camels-hair brush charged with a solution of gelatine over the aperture, then allowing it to become hard. They should afterwards be polished by gently rubbing with an oiled cloth. By this method, terebene, eucalyptus oil, creasote, cascara sagrada, cod liver oil, and castor oil can be readily swallowed without tasting the drug. Flexible capsules are now also largely used for administering the ingredients contained in Blaud's pill, with various combinations. For administering solids, cylindrical capsules are made of thin, but firm, gelatine, with a cap, or top, which may be removed to insert the drug. After filling, the cap is replaced and the joint sealed with a solution of gelatine.

Compressed Tablets.—This method of administering drugs in a dry condition, introduced to this country from America, is now very largely used. Tablets are generally manufactured on a large scale. They may be composed of a single drug or a combination of several, mixed with some inert adhesive powder and compressed into a small lenticular disc. There are several varieties of hand machine in use, which are fitted with steel dies, for making one, three, five, and ten grain tablets. The plunger used as a compressor is worked by a lever. The powdered drug is compressed between two concave surfaces, and so formed into a tablet. The three main points in tablet making are: First, to carefully regulate the pressure; second, to ensure proper cohesion of the particles of substance under compression; and, third, to prevent adhesion of those particles to any part of the machine. The material to be compressed should not be too finely pulverised, but be reduced to a granular powder. It may be prepared by simply damping with ether or alcohol, or by rubbing up with a little powdered soap and afterwards passing through a sieve. In other cases, powdered gum acacia, starch, or sugar, are mixed with the drug.

Enemas.—The preparation of enemas in most cases comes more within the province of the nurse than the dispenser. The amount of fluid administered by this method varies from two to thirty ounces for adults. The manner of preparing the ordinary enemas of gruel, soap, olive and castor oils, we need not describe. The Pharmacopœia includes five medicated enemas, viz., aloes, asafœtida, sulphate of magnesium, opium, and turpentine. The enema of aloes is composed of aloes, carbonate of potassium, and mucilage of starch, and is simply made by rubbing the solids together in a mortar and mixing with the mucilage. Enema of asafœtida is prepared by rubbing thirty grains of asafœtida in a mortar, with four ounces of water, added gradually, so as to form an emulsion. Enema of sulphate of magnesia is made by dissolving one ounce of sulphate of magnesia in fifteen fluid ounces of mucilage of starch, then adding one fluid ounce of olive oil, and mixing well together. Enema of opium is simply made by adding half a fluid drachm of tincture of opium, to two fluid ounces of mucilage of starch, and mixing. Enema of turpentine is prepared by shaking together, one fluid ounce of oil of turpentine, with fifteen fluid ounces of mucilage of starch.

Hypodermic Injections.—The preparation of hypodermic injections requires the greatest care and accuracy. They should always be freshly prepared when possible, or if kept, they must be preserved in well-stoppered bottles away from the light. Distilled water should always be used. In most cases they are simple solutions of the active agent in water, but sometimes a preservative or solvent medium must be added. One per cent. of pure carbolic acid is usually added to the injection of ergotine, while the injection of physostigmine is prepared, by rubbing down the extract with rectified spirit, then adding powdered gum acacia and water.

### Post-Graduate Clinics for Nurses.

By a Trained Nurse.

### XVII.—THE GENTLE ART OF RESTING.

I have often been struck by the fact, learned amid the many philosophies which hospital life affords, that as a general rule the woman who is a good children's nurse is equally successful in the care of irritable and nervous adults. By far the most admirable types of "rest-cure" nurses I met with in the United States during the time I was in charge of a Weir-Mitchell hospital were those who were fond of children, and who had been trained in children's hospitals. And the explanation as to the reason a good child's nurse will be acceptable to the nervous patient is sufficiently obvious, for babies and little children are exquisitely sensitive—just as are patients suffering from nerve exhaustion, brain tire, and physical prostration, to the temperaments of those around them. "One of the chief reasons," once said a very famous "nerve-rest" doctor in my hearing, "why it is so important to remove nervous and 'rest-cure' patients from their home surroundings in order to get them into the best conditions conducing to their cure, is the fact that we thus bring them into contact with persons whose temperaments differ from their own. To be surrounded by persons of different diatheses and temperaments seems to have as beneficial an effect on the mental state as change of air and scene has on the bodily condition. You must have noticed," he continued, "how some members of a family get on to one another's nerves, with a resultant want of harmony in their home relations. Often I trace this entirely to 'a matter of temperament.' And when these conditions are present in the case of one of my rest-cure patients, I insist on his or her removal to private lodgings, or a home hospital where different and fresh temperaments will at once exercise a soothing and beneficial effect."

Had these sentiments been expressed by a less eminent authority in his special branch, I might have hesitated to quote them. But, weighted by his wisdom and knowledge as to all sorts and conditions of neurotic developments, they seem to contain the explanation of many of the phenomena otherwise inexplicable which one has noticed during one's nursing experience. There certainly is no better solution of the instances so often cropping up of patients naturally most gentle tempered and amiable developing in the course of their illness an undreamt of fierceness towards those members of their family who take on themselves some duties of the sick-room. And it is by no means uncommon on a nurse arriving to take over a private case to be met on the threshold of the sick-room by a distracted relative, who says, "I am sure, nurse, I do not know how you will be able to manage him. We cannot do anything with him, for he refuses his food and medicine and will not let us do the simplest thing." Nurse at once sets her lips, calls all the strength and resources of her character to the surface and prepares to meet a terrible fierce creature, possessed of all the dogged obstinacy of fifty persons concentrated into one individual. But as a matter of experience such patients are often most easy of management and prove docile to a degree. Cases such as these are doubtless matters of temperament—a theory which we can support on very eminent authority—and through a clash of temperament the patient and his friends have become criss-cross and out of harmony one with the other. The success of the nurse in such a case is explained satisfactorily by the friends in some such way as this. "Of course it is the training that does it. She learns in hospital just how to manage sick people." Which is true to a certain degree. But no amount of training helps some nurses to "get on" with sensitive and irritable patients, while others possess the faculty naturally from the day of their first entrance into hospital. If a nurse does not possess a natural soothing temperament, a certain amount of negativeness of manner and feeling may be cultivated. We know there are some men and women to whom all babies naturally "take," and these people can soothe and rest a naughty wayward child as if by magic. And happy the nurse who possesses this motherly spirit, for it is the mother element which draws and irresistibly attracts all living things from the baby animal upwards. There are many other excitable and nervous persons devotedly fond of children, from whom all infants turn with an instinctive dislike. Most of us in our nursing experiences have met with examples of that type of nurse whose mother instinct teaches her to charm away the irritability and fractiousness of a sick child. And it is the real woman nature which teaches such a nurse how and when to rest her sick patient, though, failing such natural knowledge, she may be helped to the same end by learning how essential it is in the nursing of every case of illness to set apart regular hours day by day when amusement and entertainment is banished from the sick-room with a rigidity worthy of the old Puritan spirit! The patient, free from artificial stimulation, finding no cheerful element provided for him, in self-defence and from sheer boredom will seek that sleep whose effect contributes so materially to his convalescence. And to be an accomplished mistress of this gentle art of resting is to attain to one of the chiefest and most desirable of all the faculties needed in the delicate art and craft of nursing.

It has always seemed to me such a charming idea to be the eyes, the hands, and the thinking power of one's sick patient, and altogether to constitute the agency whereby he is led back to his normal health and strength. In the worst stages of an illness, not only has all the thinking to be done by the nurse, but she has also to contrive even the digestion of his food by the cunning care with which she selects just those articles of diet which he can best dispose of, and which will most materially benefit him. And in touching the question of food we reach another point on which rest has a most important bearing. I have so often noticed in private practice—and it is a custom honoured by observance in hospital —that the permission given by the physician for the patient "to get up a while" is so often used at the wrong time. That is to say, exertion is allowed when rest should be taken, owing, perhaps, to want of understanding on the part of the nurse of the real and important principles underlying rest. And it is such principles as these that those who care for the higher art are learning day by day, and are ever pursuing that wisdom and knowledge which it is so difficult to learn altogether from books and from the experience of others. In my earlier nursing days I used, as a matter of course, and in accordance with the custom of each ward in which I received the various chapters comprised in my whole training, to get convalescing patients "up". directly after dinner. The real reason why this is so universal a custom in hospital is so that the patient may be dressed and the ward "tidied" by two p.m. But it would be difficult to find a custom which comprises in itself so much bad nursing and bad dietetics. It is supposed to be good "ward discipline" that everything should be spick and span and in perfect order by a certain definite time. And so as to comply with this unfortunate tendency to put discipline before nursing— and this tendency is terribly strong in some training schools —throughout the hospital in which I received my knowledge of the A B C of nursing we used to "get up" our convalescents for the very first time after a long illness at that time which best suited the routine of the ward, rather than at the particular time which was best for the patient.

### On the Importance of Little Things in Nursing.

By G. Stanmore Bishop, F.R.C.S. Eng., Hon. Surgeon Ancoats Hospital.

In these days of Jubilee nurses and hospital training, there is great danger to many of the nurses themselves, that whilst they are taught anatomy, physiology, and kindred subjects, which may or may not be useful to them in after life, the small details of their work, which make all the difference as to their actual value as nurses, may be neglected or treated as of no moment. Not long since, I came across a nurse who had all the latest tests for urine at her fingers' ends, but who incidentally showed the worth of herself as a nurse by referring to one of the surgeons for whom she worked as an intolerable faddist. "He was so cross the other day," she said, "because the water I was using for irrigation was rather cold." And I cannot but think that this tendency to sneer at the importance of comparatively little things is on the increase.

When one comes to specify these things, of course, nowadays, one's first thought is of cleanliness—surgical cleanliness—and more especially in little things, and on slight occasions. Most nurses carefully prepare themselves for an operation—though I have known a hospital in which it was only thought necessary for the chief sister to do this; whilst the junior nurses, whose duty it was to look after the sponges, towels, &c., were allowed to act as they chose. But, putting this aside, do all nurses carefully asepticise their hands when they irrigate a vagina, for instance? When they pass a catheter? Are they careful to cleanse the vulva and the meatal orifice before doing so? About three months since I removed an ovarian cyst, and the patient recovered perfectly, except for a tiresome cystitis. Later I found out why that cystitis occurred. The patient told me that the nurse made two attempts with the catheter before any urine passed; that when she drew it out the first time she found the eye of the catheter plugged with some whitish material. Whether, in the first instance, she passed the instrument into the vagina or not I cannot say, but I asked what she did then. "Oh!" said the patient, "she blew down the catheter and passed it again, and then the water came all right." "Did she wash the catheter in any way first?" "No; she only blew down it." So the nurse had as carefully inoculated that patient's bladder as if she had done it of set intention.

There is a marvellous tendency on the part of many, surgeons and nurses alike, to mistake the letter of their instructions for the spirit. Certain measures, certain lines of action, even certain drugs, become fetishes. Antiseptic surgery in its earlier years had to struggle against this even more than against frank opposition, strong as that was at one time. When carbolic acid was chiefly used many men were perfectly content to dip their fingers in five per cent. carbolic solution before commencing to operate, and when suppuration followed, expressed their total inability to understand it "since they had operated under antiseptic conditions." Now it would seem to be as hard to convince some nurses that it is not enough to thoroughly asepticise their hands, if immediately after, or at any time before the work to be done is over, they smooth the patient's hair, arrange the blankets, move the furniture, or in any other way allow septic or doubtful objects to come in contact with them. Such a nurse will wash and scrub her hands in turpentine, spirit, carbolic, sublimate or biniodide lotion, or whatever antiseptic happens to be favoured by the surgeon in whose ward she was trained, and then lift the patient, shut the window, go out and bring in a chair, a pillow, or even, as I have seen, coal for the fire, and then proceed to squeeze out the carefully-prepared sponges and hand them for use in an abdominal section if she s not watched and stopped, perfectly serene in the consciousness that she has once gone through the accepted formula of disinfection. This can hardly be because she has not been taught the reasons for such a formula, since, nowadays, the theory of asepticism is one of the first things drilled into surgical nurses, and there exist several very complete little handbooks on the subject, written specially for them. I can only imagine that such nurses look upon their work as dependent on a series of arbitrary formulæ which they have to learn, and which, when learned, are to be carried out mechanically. I say "such nurses" because they must be carefully distinguished from many whom I have had the pleasure and advantage of meeting who do their work with an intelligence and care which proves unmistakably that their whole heart is in it; but, I repeat, this is not true of all, and one careless or indifferent nurse who does not realise the exceeding importance of these "little things" may ruin the work of all the rest.

There were good nurses before the days of antiseptics, from which we modern surgeons and nurses are too apt to date the commencement of surgery. Both surgeons and nurses counted their triumphs when carbolic solutions were unknown and perchloride of mercury was only a poisonous salt, and it is worth while to consider how these were obtained and whether some of the things done then are not in danger of being ignored whilst our minds are so persistently bent upon asepsis as the one gospel of surgery.

And, most of all, I think that nurses were more careful to keep their patients warm. I have seen a modern hospital-trained nurse place a patient in a cold macintosh sheet before vaginal irrigation. I have seen nurses drag a patient with raised temperature to the edge of the bed and place her so that the legs and thighs, quite unprotected by long thick stockings or anything of that kind, were half out of bed and wholly exposed to cold air. Modern surgeons are now laying, and with reason, more stress upon the conservation of the animal heat of a patient during an operation, especially an abdominal one, by the use of hot water reservoirs upon which such patients may lie, by swathing the chest and limbs in hot cotton-wool, &c., trusting almost as much for the successful result to the preservation of all the resistive power the patient possesses as to the avoidance of bacterial infection. But surely a good nurse would do this instinctively, without the need of continual reminders, were it not that her whole attention is directed to aseptic precautions, and that even if she is not taught that success depends upon this, and this alone, it yet bulks so largely before her mind that other equally important matters are forgotten or treated as of no importance. And if this is true of an operation, it is equally true in the minor work of dressings, irrigation, insertion of tampons, giving of enemas, &c. It is the easiest thing in the world when a patient has a heightened temperature to give a fatal chill during the slight exposure necessary for enema giving—still more when that exposure is neither slight nor short. But to a nurse whose mind is full only of gauze and biniodide of mercury, mingled with views on the diazo test, such matters are only often apparently too trivial for attention. The older nurses, at whom these young ones sneer, having none of the help that asepsis gives, won their hard-earned successes by constant and painstaking attention to things of this kind, and there can be no doubt whatever that the best results are to be obtained by equal care in both directions.

And almost the same remarks apply to the question of feeding. Any surgeon of any experience will know of one or two nurses, rarely, I fear, more, upon whom he can rely for this purpose. I have seen a case going steadily downhill whose prompt recovery dated unmistakably from the date when the nursing was changed. The previous nurse fed her patient strictly according to a cast-iron rule, and when food was refused, as frequently happened—was perfectly content to remove what was provided, and calmly report to that effect to the surgeon. The new nurse was not so easily satisfied. With some knowledge of cookery herself, and still more sense of what was appetising and what was not, she saw that her patient was not "overfaced" with quantities too large at any one time; that when served it was hot, and agreeable to the eye; that there was sufficient variety; that various things were tried over and over again in various ways; that dirty cups and plates were never left about to disgust and depress the reawakening appetite; and in a hundred "little ways," and by a hundred "little things," she turned the tide once more towards recovery and saved the patient. Both nurses were hospital-trained nurses, but the actual worth of the latter was at least five times that of the former.

A nurse should look upon herself as a sentinel on outpost duty. In time of war the safety of an army may depend upon the keen vigilance and the noting of "little things" of one man. Carelessness or indifference on the part of such a man at such a time is criminal, and is rightly treated as such. Prolonged watching, with every sense alert for danger from every source is impossible, and all nurses should have a full and generous allowance of sleep and recreation. I dread, from bitter experience, of all things the woman who boasts that she never had her clothes off for three days and nights. The very boast itself proves her incompetent and untrustworthy, since no living being can keep up to the requisite pitch for any such time. Let the rest, relaxation, and feeding be ample, but when on duty nothing which will help towards recovery; nothing which may mar, or even hinder, it should be too much or too trivial to escape her attention. We are always at war, at war with subtle, infinitesimal, but all-potent agents for evil. After all our efforts we may fail, but even in defeat we may at least have the proud knowledge that we have done all that human power is capable of doing; that the evil result is due to no fault or omission of ours. To be sure of this we must, above all, be able to trust our sentries not merely with our lives, but with what is of far more importance—the lives of others—those who in their sorest need have trusted us. Englishmen have always looked upon this as their most sacred duty, and it should never be possible to say that Englishwomen fail to reach, or even exceed, the high standard set up and kept unscathed through many generations of mere men.

### Appointments.

MATRONS.

WEST BROMWICH DISTRICT HOSPITAL. — Miss Aileen Moriarty has been appointed to fill the post of Matron at this institution. Miss Moriarty was trained at the Borough Hospital, Birkenhead, and has since had the charge of wards at the Adelaide Hospital, Dublin, and at the Hospital for Sick Children, Pendlebury. More recently she has held the appointment of sister matron at the Rous Memorial Hospital, Newmarket, and that of matron at the Manchester Consumption Hospital.

WEST BROMWICH INFECTIOUS HOSPITAL. — Miss Ellen Ogden has been appointed matron at this hospital. Miss Ogden received her training at the Salford Royal Hospital, where she remained for four years. Her last appointment was at the Eastern Hospital, Homerton.

### Dr. Rentoul's Bill.

WE have received from Dr. Rentoul the draft of a Bill which he has devised, entitled "An Act to provide for the Training, Compulsory Registration, and Supervision of Midwifery Nurses." This, he suggests, that we should publish, but as it covers ten closely printed foolscap pages, we feel quite unable to accede to his request.

Broadly, the proposed Bill would establish an order of beings called midwifery nurses. The term midwifery nurse would mean a person who acts "as a midwifery nurse in all cases of labour under the direct supervision of a registered medical practitioner, and who nurses mothers and infants under such supervision as is laid down in this Act," &c. Such persons are to comply with certain regulations, are to be registered, and are to be subject to the disciplinary powers of the Divisional Midwifery Nurses Board; they are not to receive any certificate or document of competency, but are to be licensed annually by the local sanitary authorities, paying each year a fee of three shillings and sixpence.

Provision is made for registering existing midwives as midwifery nurses, and a further arrangement is made that such midwives as already possess certificates shall not be prejudiced. Such being the Bill, we naturally inquire where comes in the midwife, the woman who attends confinements by herself and not under the "direct supervision of a registered medical practioner." Well, as a fact, she does not come in at all.

It is provided that no one shall take or use the name of midwifery nurse unless she be registered, and a penalty is imposed on any person who, not being registered, shall take or use such name, "or who shall use the word midwife." But are we to believe for a moment that the use of an ancient word like that of "midwife," and the occupation of which it is the name, will be abolished by a side wind like this, a mere alternative paragraph in a Bill brought in to deal with that very different person the "midwifery nurse?"

To comprehend fully the oddity of this Bill it must be distinctly understood that the midwifery nurse is a totally different being from the midwife. Although it is left to the Divisional Board to draw up regulations as to the education and examination of midwifery nurses, it is laid down as essential "that the course of instruction and of examination shall not include the theory or practice of medicine, or of surgery, *or of midwifery*, or of pharmacy or dentistry, or any part or branch of these subjects."

Was ever such a giving of stones for bread! We have in our midst a set of women known as midwives who are in the habit of taking charge of women in their confinements. In consequence of the evils incident on the ignorance of many of these midwives, and the absence of control over all of them, a strong desire has arisen for an Act under which they shall be educated and registered.

In place of this Dr. Rentoul puts forward a Bill for registering a set of persons, midwifery nurses, who, while not nurses in the accepted meaning of the term, will equally certainly not be midwives. Although the origin of the whole agitation in favour of legislative interference with midwives is the low standard of knowledge current among them, the substitutes for midwives which Dr. Rentoul proposes are, by the provision of his Bill, actually refused both education and examination in the theory or the practice of midwifery, or any part or branch of the subject! We can hardly believe that anyone will take this production seriously; our only interest in it lies in the fact that it is put forward by a direct representative of the medical profession, and that the profession can hardly escape a share of the discredit which must attach to it.

### The Victoria Commemoration Club.

THE Secretary of the Victoria Commemoration Club, 29, Southampton Street, Strand, asks us to remind nurses that the privilege of joining without an entrance fee ceases at the end of June. The club has made a successful start, and we understand from Miss Thomson that there are now between two and three hundred members.

### A Letter from Greece.

A correspondent writes from the English hospital at Calchis: We have here in all nearly 90 beds, so there is plenty tobe done. The hospital consists of two blocks, each having four wards and a room in each block for the nurses' accommodation. There are several severe cases, some of the worst being medical, but the chief work consists in dressing the wounded. The bullets in most cases have passed straight through the parts hit and left fairly clean wounds, but in some instances they have remained in, and dirt and smoke have choked the wounds and made very ugly work. We have not had many amputations; considering the number of bad cases there have been wonderfully few.

It is quite astonishing how much pain the men seem able to endure. In cases where no anæsthetics have been given they have gone through their operations with really marvellous courage. We have an Italian boy here, aged 18, who was shot through the lower part of the abdomen, the bullet passing out behind. He is in a most critical state, and we fear now there is no hope of his recovery, for signs of peritonitis are present. He came to us from the military hospital. Two very bad medical cases, one dropsy and acute diarrhœa, the other obscure, emaciation its most striking feature, occupy one room. They are cases that each need a special nurse really, so with thirty-eight other cases there is enough to keep two of us going all day and one at night. There are two nurses on day duty and one at night in each block, with two orderlies by day and one by night.

Now the work is not really so heavy as when the rush of patients came in from Domoko, but there is a great deal to do, and we expect to have some more wounded sent down from Lamia. We shall probably remain here about two months. The sisters from Karavassera are, I believe, going to stay at the hospital at Patras, where many of the wounded will be taken. . . .

Our great trial is the language. Not being able to understand or make yourself understood is a great trial in nursing, especially when giving instructions to the orderlies and sending them for things one needs. It is hopeless now and then, especially at meals, when there are so many different diets to be seen to.

The patients here have coffee and bread at six a.m., lunch of meat and wine at eleven a.m., coffee and bread at three p.m., and meat, soup, and wine again at six p.m. They drink the wine of the country flavoured with reisin. It is not nice at first, but one gets to like it in time, and it is very wholesome. The cry all day is for "nerō" (water). They drink any amount of it. The other day the supply ran short, and though it was only for a very few hours, it made one feel as if the hardships of war were not over. Taking the patients all round, they are grateful and very patient, and their wants few and easily satisfied. At Karavassera the work was done by Greek doctors; here we are all English, which makes things much easier.

There are a good many points of interest about this town, one being the prison to which most of the long-sentence criminals are sent; it is said few survive their time of punishment. Some of the sisters have been to see it. The heat has not so far been oppressive, except in the middle of the day, when everyone should follow the custom of the country and take a siesta; the evenings are cool and fresh. This place is twelve hours by boat from Athens, an interesting journey. Greece is not up to date in the matter of boats and trains; no one ever seems to know when they may be expected to start or arrive. Considering the modern history of the country perhaps one must not expect much.

We do not expect to have any further rush of work as things are. The armistice is said to last 17 days, but we suppose it really means that the war is over.

### Be Loyal.

(By a Hospital Matron.)

The sentiment predominant at the present moment is loyalty. We are stirred by current events to feelings of devotion to our Queen and country, and it may not be amiss to consider the subject from a nurse's point of view.

I take it that we, as nurses, should be loyal—First, to God; secondly, to our country; thirdly, to our employers.

To be loyal we must be faithful and true, devoted, attached, obedient, and trustworthy; we must understand our duty, and be willing, if need be, to sacrifice our own interests in following it; and we must thoroughly comprehend to whom our allegiance is due. We live in an age of excitement and hurry—there is a tendency to rush off into anything and everything that will bring variety and change into the monotony of the daily round. Few pause to consider consequences or to weigh motives, and a catchword may carry the day. Thus we hear of women hastily relinquishing their work, and flinging themselves into alluring schemes, eager for self-advertisement, dazzled with visions of possible aggrandisement, and blind to the fact that by their impetuous action they may be doing an injustice to their employers and neglecting their immediate duty.

Loyalty to our own Queen and country rank [illegible]xt to our individual duty to God, and who knows how soon we may be called to play the part of patriots. No women could give such direct national service as fully trained nurses in the time of war, and there is not a shadow of doubt when and if the call comes that the supply of volunteers will be greater than the demand. But until this happens are we to forget our third duty—loyalty to our employers? Are we to imagine that anything can be higher and more noble than the honourable performance of each day's work—the never-failing giving of our best? Surely the nurse who works quietly and devotedly in her own sphere is worthy of as great commendation as the blatant recruit who rushes into new enterprises, assuming unknown responsibilities for the sake of questionable notoriety and spurious glory. For the free-lances of the nursing army, for those who know no employer and are bound by no ties, it matters not—they may come and go as they like, and may pin any colours in their bonnets but let others pause and think and consider to whom they owe their allegiance.

It is difficult to over-estimate the value of loyalty to those over us in authority. Oh! if we could each of us be absolutely true to our superior officers. How easy would it then be to work, how smoothly the wheels would run, how responsibilities could be shared, and burdens lightened. It lies with each one of us to see that we are devoted and attached to those under whom we serve, even though there be no sound of popular applause in our ears, and our only satisfaction the knowledge that our conduct has been honourable, and that we have in no wise failed in fidelity.

### Novelties for Nurses.

THE PERFECT SAUCEPAN.

The duplex boilerette is the best form of saucepan we know. We have come to this conclusion after giving a long and careful trial. Boiling over is avoided, and the boilerette requires no watching owing to the safety steam valve. There is an inner saucepan to contain the food to be cooked, the outer receptacle being full of water. The food retains its flavour, and cooking is rendered easier in every way by its use. We recommend it to every housekeeper. It can be obtained at the Duplex Works, North Newington, Banbury.

### A Book and its Story.

OUR ENGLISH CATHEDRALS.

OUR last review dealt with the first four of a series in course of publication descriptive of our English Cathedrals. The series of dainty little booklets* comprises now, in addition, the histories of Canterbury, Salisbury, Gloucester, and Norwich Cathedrals, volumes which fully maintain the standard of excellence attained by their predecessors in this series, both as to letterpress and illustrations.

The history of Canterbury Cathedral is written by Dean Fremantle, whose pen touches chiefly upon the most salient points of interest about the building, which interest, according to the author's words, "increases with the increasing study of its history," concerning which, from time to time, new discoveries are being made which throw fresh light upon its antiquities and its architecture. No English Cathedral is better known than Canterbury; the city itself also possesses associations peculiarly its own. Here was the first home of Anglo-Saxon Christianity. When Augustine passed towards the city, as described by the Venerable Bede, "with his little procession headed by the monk carrying a board on which was a rough picture of Christ, and a chorister bearing a silver cross, his heart, no doubt, beat high with hope, but his hope would have grown into exultation could he have looked forward through the centuries and beheld the magnificent Cathedral which was to spring up where his episcopal throne was fixed," and the energetic and varied Christian life which has evolved therefrom. The early history of Canterbury is but little more interesting than its succeeding, and it has found a sympathetic biographer in the Dean, whose former close association with the Cathedral enables him to speak with authority on all questions appertaining to it.

The history of Salisbury Cathedral, as told by Dean Boyle, shows that it cannot boast of the same antiquity as can Canterbury. It was close on the eleventh century when the first small church of Old Sarum was consecrated. Its evolution can be steadily followed, and the brief records of the lives of the Bishops connect the history of the Church with the annals of a great period, when the constitutions of Clarendon were keenly debated, and the quarrel between Thomas à Becket and Henry II. was gathering strength. The Cathedral of Old Sarum, though small in proportion, had some beauty of its own. William of Malmesbury said that its builder had cause to say, "Lord, I have loved the glory of Thy house," "but still one reason," writes its present chronicler, "for the removal to New Sarum was given in the allegation that wind and storm sometimes prevented the hearing of the service. In the interesting document which records the foundation of the new Cathedral in the year 1220, although there is much that is legendary, there is enough of real historic value to enable us to realise how all the difficulties were overcome," and the structure gradually grew into the beautiful form of its later date. Forty years intervened before the Cathedral was actually finished.

In the year 1280, for some unknown reason, the Cathedral was re-dedicated; "but one of the great glories of Salisbury, the spire, was only added sixty or seventy years after the rest of the building was completed. . . . Much has been done to strengthen the masonry in recent years, and important precautions have been taken to secure as far as possible the safety of the grand memorial of the Middle Ages, placed by Macaulay in his famous chapter side by side with the towers of Lincoln. The Cathedral of Salisbury certainly stands out as the finest specimen of the Early English style. Dean Boyle pays all tribute fitting to the subject of his theme, and the interest of the Cathedral, externally and internally, as epitomised by him shows it to be one of no ordinary nature.

"Norwich Cathedral," Dean Lefroy writes, "has weathered eight centuries of our national life. It has survived some of the most violent changes in our social, political, and religious history. It has witnessed scenes which would inspire the imagination of the poet, the genius of the painter, and arouse the sorrow of the saint. . . . To rightly understand the important position this Cathedral attained in our national life, and that its greatest institution and most influential moral agency attracted even Royal attention, and enjoyed Royal sympathy, we must remember that Norwich was once the second city in the kingdom. . . . The sacred building is rich in architectural beauties. The Norman apsidal chapels, the processional walks, the seven sacraments font, the rare specimens of transition Norman, the tower, the spire, which," says the Dean, "will, it is hoped, abundantly justify the claim here made for it."

Speaking of the actual building of the Cathedral, the Dean tells us that, for the vindication of his honour, Herbert de Losinga is said to have engaged to Pope Urban that he would build a cathedral at Norwich. Probably the Roman sacrament of penance was never so fertile, in any age or place, as it was when, the first stone of the great Norman Church was laid, which is more utilised to-day than at any period in its history. Herbert de Losinga's work was carefully thought out, and personally superintended by himself. "From the first he planned his work, and while he accomplished the greatest section of it, and others completed what he had left undone, yet it was his design which they developed and completed. This has given to Norwich Cathedral that impression of unity which marks it off from other inspiriting constructions. Norwich Cathedral represents some of the loftiest expressions of religious fervour. It commemorates many of the saddest incidents in our national life. It conserves the unstinted consecration of time, talent, treasure, and love, to the adoration of God and the elevation of man, and it has presented to the Most High the sacrifice of praise in varied forms for well-nigh thirty generations of human life."

The architecture of Gloucester Cathedral is carefully dealt with by Dean Spence, and Mr. Herbert Railton's illustrations certainly justify the writer's enthusiasm of the stately beauty of the edifice which he describes. At first glance, Dr. Spence remarks, "a stranger . . . who knew nothing previously of its story, would hesitate before he called it a great Norman church. The lordly Perpendicular tower, if less vast than the mighty mid-tower of Lincoln—the grandest of our English towers—is certainly more graceful. The long line of Decorated windows looking into the college green, the huge choir window, the matchless Lady Chapel at the east end telling of the closing year of the fifteenth century—all these prominent features would indicate rather a Perpendicular and Decorated than a Norman pile. Only, when the stranger began to look more closely into the details of the exterior of the great church, he would see signs of an older school of thought." One of the prominent characteristics of the Cathedral are the doors—magnificent examples of Norman wood and iron work; indeed, these are among the most ancient examples of any extant. Another striking feature in the lordly pile are its windows, among which is the "Cressy" window, a memorial of the national victory. The finest cloistered walk in England is here; it has been said there is none finer in Europe. Here also is the earliest example known of fan-tracery vaulting, and, as has been said, it is one of the titles of honour of the monk-architects of Gloucester Cathedral that they first devised this lovely and peculiarly English form of ceiling.

* "Canterbury Cathedral," Very Rev. Dean Fremantle. "Salisbury Cathedral," Very Rev. Dean Boyle. "Norwich Cathedral," Very Rev. Dean Lefroy. "Gloucester Cathedral," Very Rev. Dean Spence. (London, 1897. Isbister and Co., 15, Tavistock Street.)

### Nurses and Social Grades.

THERE has been observable of late a growing and regrettable tendency amongst a certain class of people to narrow the nursing calling into one from which it is desired to exclude all women save those who can label themselves "gentlewomen." Sneers are cast at hospitals which still wisely and rightly accept as probationers superior women of the domestic servant class, and it is taken for granted that in days to come "ladies" will have entirely ousted their more humbly-born sisters from the nursing field.

Now this is a question which ought to be honestly faced by all who have the honour and welfare of nursing at heart, and considered in all its bearings. Is there really to be room for none but those who can produce a certificate of gentility in the hospitals and sick-rooms of the future, and if so, in the name of common sense and reason, why? Surely the most experienced and broad-minded matrons of to-day will have but one reply, and that is that the profession of nursing can not afford to cast off the many intelligent, hard-working, and fairly well-educated women who now find employment and do good work in its ranks.

The reasons for coming to this conclusion are not far to seek. Amongst nurses there must ever be drawn a distinct line of division between those who pass through their hospital training and rise to the high places in their profession, who by character and position are fitted for administrative posts, and whose special gifts will make them excellent heads of wards and superintendents of institutions, and those others who throughout their lives will be content with subordinate posts, and will find their proper sphere in private nursing, or as staff nurses under a sister. Not to these will, or should, be given the responsibility of training others, of giving lectures or holding classes, nor will they be placed in positions which can only be adequately filled by gentlewomen, but it is idle to say that there is no room for them in the world of nursing. We shall all agree that it takes a lady, in the truest and widest meaning of the word, to make a successful ward sister or head of a training school, not necessarily because her aptitude as a nurse is greater, but because the qualities needed for such a post are something more than go to make a nurse. None the less will it be readily acknowledged by most hospital matrons that amongst the best and most reliable nurses on their staffs may be counted numbers of those who would make but a poor figure in the unnecessarily and absurdly stiff theoretical examinations which are, unfortunately, becoming more and more the fashion at some institutions.

Many medical men, and those of the widest experience and highest authority, will confess that for private nursing they prefer the much-abused "housemaid" nurse, and that is a view which will be cordially echoed by those of the public who have suffered at the hand of her social superior, the "lady nurse." It is a self-evident fact that ladies do not make the best private nurses, and one which few who have had experience in the management of a private nursing institution will deny.

There is, then, it is clear, room for the intelligent and educated daughters of the farmer and the artisan, the girl who has "been to service" and gained there practical experience in the sweeping and dusting and other domestic details which must be mastered by every really efficient nurse, as well as for the lady born and bred, whose social qualifications and highly-trained mind will fit her for the highest posts in the profession. The point is that the truth and the justice of these necessary distinctions need to be honestly realised and acknowledged. There are, one is glad to know, many women who cheerfully recognise their own limitations of position and intellect, and only wish to do what they can to lessen the sum of human suffering. And who will dare to say that this may not be done by the trained nurse who has been a housemaid as well as (and in some cases better than) by the trained nurse who is the daughter of a peer?

### Everybody's Opinion.

[Correspondence on all subjects is invited, but we cannot in any way be responsible for the opinions expressed by our correspondents. No communication can be entertained if the name and address of the correspondent is not given, or unless one side of the paper only is written on.]

### NURSES AND CYCLING.

"IRISH QUEEN'S NURSE" writes: Is it not a shame that nurses, who are the hardest working of all women, cannot amuse themselves like other people without drawing down an avalanche of wrath from public opinion? If the two nurses in Guy's uniform who were seen "with a bike on London Bridge platform" by your correspondent had been idle society young ladies of no definite occupation whatever no remark would have been made. It has been said nurses should go out of uniform when amusing themselves. The fact is, we have so short a time off duty that it is scarcely worth while to go into mufti, as every nurse knows well. Hospital nurses in wards all day must naturally enjoy cycling; it is less fatiguing than walking, of which they get plenty when on duty, while to a district nurse a bicycle is a boon. The reason nurses are so much to the front now in magazines and newspapers, which fact seems greatly to annoy "A Womanly Woman," may be partly due to our Most Gracious Majesty, who has taken so much interest in our profession. One does feel indignant at the criticisms that are constantly passed upon us and our deeds; therefore I cannot refrain from sending this protest.

### "NARROW-MINDED NURSES."

"A NURSE" writes: Having seen an article on narrow-minded nurses, I should like to give my opinion on the narrow-mindedness of doctors, although I do not wish to class them all together; but still I think the majority are too fond of petty gossip. For instance, a nurse enters hospital with her heart and soul in her work, and works on happily, till at length some trifling unpleasantness arises, perhaps due to the nurse's own carelessness. She knows where to find a ready listener at any moment. A sensible nurse would not be so easily led, but one meets with "weak-minded" as well as "sensible" in the nursing world, as in any other profession. The kind friend listens patiently, with many a disrespectful remark, teaching the nurse to be insubordinate and disrespectful to her superior officers. She naturally sides with her eager listener, until she hears her words repeated by her charge nurse, or perhaps a probationer. Then she wonders. Surely Dr. —— couldn't have repeated what I said to him. Oh, no! A "man" would never be so mean. But, alas! too true. So I think I can strongly and safely second the writer's opinion by saying that if doctors were less fond of discussing nurses one to another, they would not only gain more self-respect, but nurses would live much happier, and so raise the whole tone of discipline which has in every other possible way a good example set. I write this from experience.

### NURSES AND CYCLING.

"HYGIENIST" writes: While I quite agree with "A Womanly Woman's" strictures, as expressed in your last issue, against women making themselves conspicuous by cycling in uniform, I yet cannot concede that because a woman is a nurse she should be debarred from the enjoyment of such a health-giving pastime as cycling—always provided, of course, that she does not do so in her official dress. I have been a nurse for the last six years, am devoted to my profession, and give myself, "heart and soul to the work." But when off duty it is beneficial occasionally to forget the sick-room and its suffering inmate, and I find that my bicycle reinvigorates me, and enables me to return, refreshed and strengthened, to my patient. Everyone must endorse your correspondent's remark that there is nothing more objectionable than an unwomanly woman; but does a liking for healthy exercise prove a woman unwomanly? Are nurses "foremost in every amusement, magazine or newspaper"? Of the first I think there are very few in which they take part; and, with the exception of a few class journals, the name of a nurse is rarely mentioned in a newspaper or magazine of to-day. Your correspondent surely exaggerates. Did the nurses of ten or fifteen years ago "get on so well" without the enjoyments of their sisters of the present time; or did they not reap the punishment of those who give themselves up, body and soul, to one enthralling occupation? Did they not rather suffer for neglecting those healthy exercises which promote the *mens sana in corpore sano*, and which make the nurses of to-day, physically and intellectually, such a splendid body of workers? I do not consider that taking part in, and having a liking for such a hygienic pursuit as cycling is "dragging an honourable profession through the mud"; and in this I think that every unprejudiced person will agree with me.

DO SERVANTS MAKE GOOD NURSES?

"A Nurse of the Servant Class" writes: Following with interest the "Post-Graduate Clinics for Nurses," I notice that the chief complaint against nurses is their want of due economy and housewifely instincts. From my own experience I can testify to the truth of this, and I think the cause is partly the modern woman, but chiefly that probationers are now chosen from a class "above servants" or the thrifty, well-to-do working class. One can scarcely take up a nursing paper without seeing an article against these, or advising nurses to keep up their studies, &c. Good. Let ladies bring their education, culture, and refinement into the profession; those of them who are fitted will secure matronships, foreign posts, and the like. We do not ask for them, only to do hard, useful work in lowly places. It is curious this highly-trained, noble-minded nurse, a product of the nineteenth century, showing the narrow spirit of bygone ages. I should like to see some American opinions on the subject. There are in every class men and women clever, capable, ambitious, and refined; in these days no profession is closed to them, and men do not clamour against it. What I specially protest against is servants being singled out as utterly unfit for nurses. I believe with Miss Nightingale that a well-trained servant is a half-trained nurse. Habits of prompt obedience, order, method, and punctuality are very valuable in a nurse. Quickness to see what is required and a spirit of loyalty and discretion in not repeating matters which come to her knowledge will be oftener found, I think, in a servant than a girl straight from the freedom of the family circle. The majority of middle-class young women cannot afford to live at home till twenty-three or twenty-five years of age; there is no employment open to them without paying a premium or giving time except domestic service. Why should they be ashamed of entering this, laying up a store of practical every-day knowledge, and improving their minds in their leisure time (which is more than nurses get). The purpose they have in view will help to keep them from the selfishness or frivolity of their companions. I wish an abler pen had taken up this subject. I am conscious of being heavily handicapped by my lack of education, which considerably increased the strain of my three years' training in a provincial hospital (250 beds). There, however, I was never in any way treated as an inferior by my fellow nurses, who were of all classes.

[We are particularly glad to publish this letter, which reaches us at an opportune moment, treating as it does of a subject upon which an article will be found in another column.—Ed. *T.H.*]

### Where to Go.

Stafford House.—The annual meeting of the Maternity Charity and District Nurses' Home, Plaistow, E., will be held at Stafford House on July 8th, by kind permission of the Duchess of Sutherland, who has also consented to preside. Tickets can be obtained from Sister Katherine Twining at the Home. The charity has a steadily increasing debt of £700 for current expenses, and the public are asked to help by sending money and clothes (new or old), and holding meetings on behalf of the charity.

### Minor Appointments.

Park Fever Hospital, Hither Green, Lewisham.—Miss Alice Maud Armstrong and Miss Alice Emily Newport have been appointed respectively Assistant Matron and Superintendent of Night Nurses at this new hospital. Miss Armstrong received her training at the Brownlow Hill Infirmary, Liverpool, and was subsequently appointed home sister at the Infirmary, Birmingham. Miss Newport was trained at the London Hospital, and has since held appointment as matron at the Smallwood Hospital, Redditch, and taken matron's holiday duty at the Surbiton Hospital.

Poplar and Stepney Sick Asylum.—Miss Emily Cowland De Levante has been appointed Sister at this infirmary. Miss De Levante was trained at the Seamen's Hospital, Greenwich, and subsequently worked as a private nurse for two and a half years. She has since held an appointment as nurse at the Shoreditch Infirmary, and has also gained experience in district nursing.

### Notes and Queries.

*Nurses' Cycling Clubs.*

(283) My nurses are anxious to form a cycling club in connection with their hospital. Can you tell me where I can learn the plan upon which other hospital clubs are conducted?—*Matron.*

If you write to Miss Nott-Bower, matron of Guy's Hospital, London S.E., we feel sure she will be pleased to tell you the lines upon which the Guy's Cycling Club has been started, and you could not have a better example.

*Army Nursing Reserve.*

(284) Please tell me the number of The Hospital in which was given an account of the "Army Nursing Reserve."—*Saphena.*

You will find the account in question in The Hospital for March 6th, under the title of "A New Military Nursing Order."

*Lockers.*

(285) Can anyone recommend a really good hospital locker, with cost and address of firm where it can be obtained?—*Hon. Sec. County Hospital, Huntingdon.*

There are many excellent varieties of lockers, and you might write to one or two firms which go in specially for hospital furniture, and ask for catalogues and sketches of those they make. Messrs. Shoolbred and Messrs. Maple, both in Tottenham Court Road, and Messrs. Debenham and Freebody, Wigmore Street, do a great deal of hospital furnishing. A very good locker, which experience has proved to answer all requirements, will be found described in The Hospital for January 27th, 1894, p. 293. It is in use at the Poplar Hospital, and is made by Messrs. Shoolbred. For price you should write direct to the firm. Whatever locker you choose, the top should be of marble, tiles, or glass, preferably the latter.

*Monthly Nursing.*

(286) I wish to become a monthly nurse, and should be grateful if you would advise me how to set about obtaining training.—*Dorothy.*

Write to the secretary of the Midwives' Institute, 12, Buckingham Street, Strand, for advice, enclosing a stamped envelope for reply.

*Village Nurse Wanted.*

(287) Can you help me to get a nurse for a small out-of-the-way village, containing about 36 houses. We want one who is a cottager herself, and who is also a midwife. Would it be any use advertising for a country-woman willing to be trained?—*Perplexed.*

We should suggest advertising in our columns if you really must start a nurse specially for so small a village. It would be better to come to an arrangement with some neighbouring institution, from which on payment of a subscription you could obtain the services of a fully-trained nurse. In default of this it may be necessary to fall back on the "Cottage Nurse" system; which, however, is but a second best.

*Home for Incurables.*

(288) Can you tell me of a home where a young man in an advanced stage of consumption might be admitted? He is destitute, but above going to the workhouse if any other institution can be found for him. —*B. F.*

We doubt if you will find any institution for the reception of an incurable case of consumption which will admit patients free. There is St. Michael's Home, Axbridge, Somerset, which is free, only members of the Church of England being eligible, but we are not sure if advanced cases are taken. At the Firs Home, Bournemouth, 10s. weekly payment is required, while at St. Andrew's Hospital, Clewer, where a limited number of such cases are taken, 12s. 6d. to 15s. 6d. weekly must be paid. At the Mildmay Home, Torquay, 10s. 6d. a week is required. You will find a full list of institutions in "Helps in Sickness and to Health" (Scientific Press, 28 & 29, Southampton Street, Strand).

*Male Nurses.*

(289) Please tell me how to become a male nurse?—*H. F.*

A limited number of men are trained at the National Hospital for Epilepsy and Paralysis, Queen Square, W.C. Apply there if you wish to go in for training.

